# Exploring the role of ranking systems towards university performance improvement: A focus group-based study

**DOI:** 10.1016/j.heliyon.2023.e20904

**Published:** 2023-10-11

**Authors:** Tayyaba Rafique, Muhammad Usman Awan, Muhammad Shafiq, Khalid Mahmood

**Affiliations:** a Institute of Quality and Technology Management, University of the Punjab, Pakistan; b Department of Industrial Engineering and Management, University of the Punjab, Pakistan; c Supply Chain and Project Management Centre, University of the Punjab, Pakistan; d University of the Punjab, Pakistan

**Keywords:** University Ranking Systems, Focus group, Performance measurement, Thematic Analysis

## Abstract

The development of multiple university ranking systems at national and global levels has been driven by increasing interest in improving efficiency in the national educational sector without compromising the demand for international standards. Global university ranking systems play an important role by providing the foundation for competing in this global era. One approach could be developing and evaluating criteria to reduce the unnecessary use of standard, less productive indicators. This study aims to systematically exploit national and global university ranking systems in terms of their indicators and relevance to national educational needs. This study uses two online qualitative focus groups with 10 participants each. The participants were purposively sampled, and the transcribed data from the focus group were thematically analyzed. Findings indicate that university ranking should include indicators like governance and digital presence, as these are missing in global ranking indicators. These findings will guide the development of a university ranking framework that policymakers and universities can implement to improve institutional performance.

## 1 Introduction

Higher Education Institutions (HEIs) play an important role in providing advanced training and education to students. They are often at the forefront of cutting-edge research and the promotion of innovation [1,2]. These institutes are essential for developing a knowledge-based economy and play a significant role in uplifting society from a social development and economic perspective [3]. In recent years, university ranking systems have been used to evaluate these institutes' quality and performance [4]. Historically, university rankings have been associated with students' decision-making and university management [5]. According to Belov, Chernova [6], ranking systems are being used for the assessment of the international performance of universities and also allow the stakeholders (students, university management, investors, recruitment heads, and teachers) to evaluate the standing of the institute based on different features like academic performance, student success ratio, H-index, the proportion of international faculty, etc. And aid them in decision-making [7].

All university ranking systems are mainly based on the league tables, which is the second name for rankings of universities [8]. These league tables differ from one ranking agency to another. These differences entail the pros and cons and the details about which university comes after the other [9], along with better choices for people helping them select suitable institutes for themselves. These rankings are becoming crucial because they evaluate and rank a particular institution [10]. However, within the current landscape of higher education, global rankings have been criticized for being biased towards the research performance of institutes. This approach often overlooks necessary indicators and objectives associated with the operations of the higher educational institutes (HEIs) that must be satisfied, such as knowledge transfer and teaching. According to Hewitt [11], university ranking systems have become more of a marketing exercise than metrics used to assess academic performance. Thus, several academics and industry practitioners have recommended developing a precise and transparent system to rate and rank the institutes based on their academic and research performance and not their marketing efforts.

Another issue with the global ranking systems is that they fail to account for the cultural aspects and broader infrastructures [12], which undermines their application and generalizability. Similar to the problems in the global ranking systems, the lack of a proper metric for assessing the institute's performance has also prevailed in Pakistan. The lack of a national ranking system has affected the decision-making abilities of Pakistani stakeholders since the one mandated by the Higher Education Commission(HEC) was discontinued in 2018 due to criticism for the absence of a transparent system [13], presenting a significant gap from an academic and policy perspective.

The relevance of the indicators used in the well-known global ranking systems has mixed results in terms of the ability to measure institutional performance. These systems are highly ineffective and cause hyperactive competition in the global higher education system [14]. Still, they make it easier for students to select the best universities [11,15].

Some studies were conducted to ascertain and solidify the ranking criteria for higher educational institutions. However, most of them have failed to identify the factors specific to the context of Pakistan. Table 1 addresses the previous studies in this domain and explains how the present study resolvs the limitations of previous research.

**Table 1:** Limitations of previous studies.

Reference	Findings	Limitations
Noreen and Hussain [16]	According to the study, Pakistani rankings are primarily based on the standard of instruction, the standard of research, the number of Ph.D. faculty, publications, and journals. It also concentrated on the supply of amenities and the appropriate and efficient utilization of funds. Among the performance evaluation factors are social integration, community improvement, outreach initiatives, international partnerships, and foreign professors and students. However, international standards include 'citations' as a crucial factor for better performance assessment which is not as emphasized in Pakistan.	The research utilizes secondary resources, which can limit the depth of understanding and sometimes even the accuracy of the results.
Rasool et al [17]	The study elaborates that very few universities involve a third party in setting their objectives regarding quality assurance and that involve outsiders in assessing the university's performance. Further, there is a shortage of personnel in most universities who are experts in quality assurance through courses and program assessments.	The research utilizes secondary resources, which can limit the depth of understanding and sometimes even the accuracy of the results. Further, the metrics are not explicitly discussed.
Chowdhury [18]	The research focused on Indian Higher education institutions and their ranking systems. It concludes that most Indian institutes adopt the Webometrics ranking framework. They emphasize the visibility of institutes, transparency, including the cited researchers, and excellence based on several top-cited papers and their rankings.	The research focused on different ranking systems similar to India's ranking system. It utilizes a secondary source of data.
Aithal and Kumar [19]	The research compares different ranking systems adopted in different regions, including China and the US. The study suggests that China's higher education institutions focus on the quality of education, faculty quality, research output, and per capita performance.	The research focuses on secondary data sources and does not include the context of several Asian countries, including Pakistan.
Guillerme [20]	The report discusses the rankings of different institutions surrounding different regions of the world. It includes the top 200 Asian institutes according to international ranking. The institutes include six institutes from India and 18 institutes from China.	The report does not include the rankings of Pakistani Institutes.

The aforementioned constraints have highlight the absence of any research endeavour that investigates and delineates the criteria for ranking institutions in Pakistan, as identified by stakeholders, while also including primary data collection. Accordingly, the following research questions are formulated for this study;

• What are the perceived university ranking indicators which can evaluate the performance of higher education institutions?
• Are there any region-specific indicators that must be incorporated into Pakistan's national ranking system?

## 2 Methods

### 2.1 Study design

Focus groups offer a cost-effective and time-efficient approach to gathering comprehensive data compared to individual interviews [21,22]. A qualitative exploratory, descriptive design was used in this study in which the participants were led through a discussion to gain insights that wouldn't be revealed without the group dynamic; as such, they were prompted to draw analogies between different ideas as the discussion progressed [23,24].

This approach and strategy were deemed suitable given that the study's objective was to gather the participants' perspectives on university ranking and performance. Consequently, focus group sessions were devised to facilitate the collection of pertinent insights from various university stakeholders [25]. The focus groups were organized into three sessions, with the initial session presenting the research scope, core inquiries, and study importance. During the second session, the participants selected and evaluated criteria and indicators derived from foreign rankings systems. Subsequently, 30 factors were chosen and consolidated into groups during the third session. A 10-min intermission was allocated between each session.

### 2.2 Participant

The participants were selected based on “purposive sampling.” Participant selection was based on their familiarity with the topic and extensive knowledge of quality management in the education sector. There were 20 participants in this study divided into two focus groups. All participants were male, aged between 35 and 60. Participants included representatives of the Higher Education Commission (HEC) of Pakistan, the Quality Enhancement Cell (QEC), senior faculty members, and doctoral scholars.

### 2.3 Data collection

Demographic details related to the current position, gender, age, years of experience, and highest level of education were collected at the outset of the focus group. To ensure cohesion between sessions, the focus group facilitator used a set of predetermined questions detailed in Table 2. Each focus group lasted between 45 and 60 min, and all recordings were transcribed verbatim and anonymized before analysis. ⁠⁠⁠⁠⁠⁠⁠

**Table 2:** Focus group questions.

Question 1	What is the relevancy of international ranking systems indicators to performance management?
Question 2	Can the performance of Pakistani universities be measured using indicators of the global ranking system? - Are these indicators suitable? - Is there any need to make changes in indicators?
Question 3	How can performance management indicators be grouped?

**Table 3:** Summary of findings emerging from the focus group discussions.

	Themes	Sub-themes	Description
1	Performance management	University ranking systems and performance	Identification of HEIs performance management based on international ranking systems.
		Performance management of Pakistani universities	The application and resonance of international standards in Pakistani institutes
2	Pakistani HEIs	Generalization of ranking systems to the Pakistani context	Relevance of ranking systems in the country context
		Environment	Regional factors influencing the performance of the institutes
3	Indicators	Indicators for performance measurement	The indicators that relate to the performance of HEIs

### 2.4 Reflexivity

The facilitator was a female Ph.D. scholar and lecturer at a government university; because of her job, she knew some participants by name and role but did not manage or collaborate with them. As a researcher, the facilitator kept participants' discussions intact during data collection and analysis.

### 2.5 Reporting

Reporting for this study was based on the “COREQ: consolidated criteria for reporting qualitative research [26]."

### 2.6 Ethical approval, recruitment, and consent

Consent, transparency, and anonymity are essential ethical concerns involving human participants. Green and Hart [27] viewed focus group sessions as extremely vulnerable conditions as the participants are required to disclose opinions in front of peers. Sim and Waterfield [28] explained the importance of anonymity and confidentiality for focus groups. They suggested that these should be enforced to protect the research's credibility and the respondents' privacy.

To ensure the ethical integrity of the research and foster a tranquil research environment, the researchers have conscientiously addressed ethical concerns and taken the following measures:

1. The respondents' consent was achieved using a consent form before the initiation of the study. It was assured that there was no coercion.
2. The respondents were communicated elaborately about the objectives and procedures of the research. They were also informed about their autonomy to leave the session.
3. The respondents were treated relatively without any bias or judgment.

### 2.7 Data analysis

**Fig. 1:**
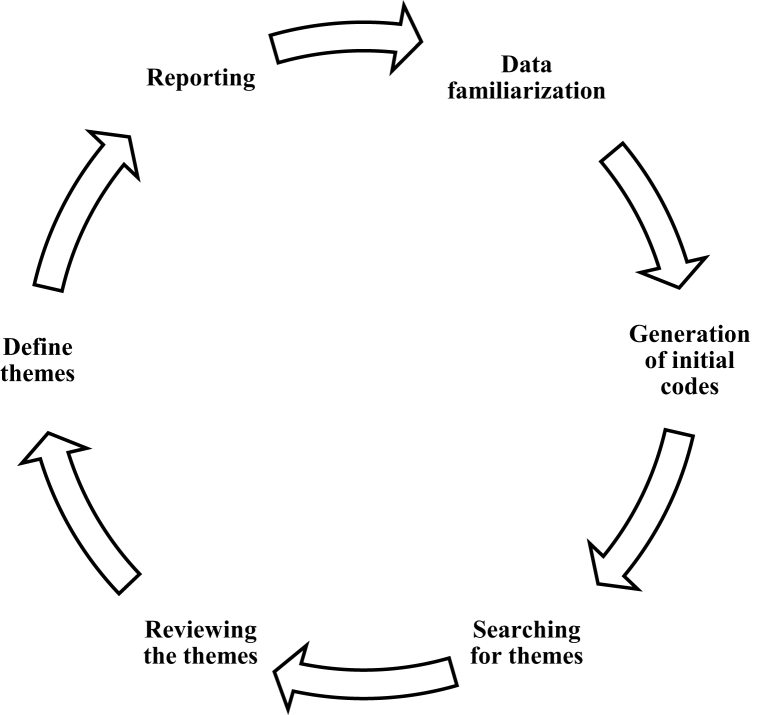
Braun and Clarke [30] thematic analysis steps.

The focus group data was analyzed using an inductive content analysis method based on the recommendations of Mayring [29], Braun and Clarke [30], and Kiger and Varpio [31]., which consisted of six stages, as mentioned in Fig. 1:

1. Data familiarization: This process includes identifying the initial codes by reading and re-reading the transcript once it's been finalized. Corresponding to the suggestions, the transcripts for each group were prepared, read, and initial themes were developed.
2. Generation of initial codes: This step involved coding the data sequentially in a series of codes reflecting the discussion's nature. Meaning units (sentences and paragraphs) were identified and labeled for each focus group transcript with a code. None of the qualitative data analysis programs were used. However, a manual color-coding process was used to identify and code themes in the transcription documents.
3. Searching for themes: In this stage, the codes were collated to potential themes, and all data was coded to related themes. In this phase, the codes within each topic were grouped into categories. Similar contents were grouped. Then, themes and subthemes were compared and grouped. The data was initially transformed into five themes (now classified as sub-themes in Table 3).
4. Reviewing the themes: A thematic map was generated, and data and codes were verified for their relevance to the themes. In this phase, the second and third authors verified the coding and thematic distribution to confirm that the thematic distribution was accurate. To ensure that the reported findings accurately reflected participants' perceptions, the research team discussed these emerging themes and subthemes and revised them as necessary [32].
5. Define themes: In this phase, as per Braun and Clarke [30], the names of each theme were finalized. After the second review, the themes and codes were regrouped into three major themes- Performance management of HEIs using global ranking systems, Pakistani HEIs and their performance management using Global Ranking Systems (GRS), and lastly, the indicators which should be used to improve the performance of Pakistani HEIs.
6. Reporting: Interpretation, data quotes, and literature were used to write each theme and subtheme [33]. The results were presented using the themes and subthemes from the analysis.

## 3 Results

### 3.1 Themes

#### 3.1.1 Performance management

The participants generally agreed that ranking systems are one of the tools that can measure university performance and help manage overall university quality. The universities use these internationally accredited ranking systems to evaluate their performance. Still, Pakistani HEIs are not performing well on global ranking systems (GRS) because the relevant staff is not trained to use those ranking systems to improve performance. Extensive discussions occurred in each focus group regarding the performance management of HEIs using GRS, and all agreed that:

“Our performance hasn't been consistent because we don't know what information to work on."

There was consensus amongst the participants in all focus groups that although HEC ranking has been used for some time to check the performance of Pakistani HEIs, there is still uncertainty regarding the specific parameters on which HEIs should work.

#### 3.1.2 Pakistani HEIs

The participants agreed on the differences between Pakistani and universities of the developed countries and how performance metrics needed to reflect this fact.

*“QS (Quacquarelli Symonds) and THE (Times Higher Education) reflect generic factors, and it is crucial to focus on regional factors as well*."

Participants agreed on developing national ranking systems, taking GRS as a guideline, and integrating national and regional factors. HEC used to assign rankings to universities, but there was a conflict of interest in assigning ratings and rankings. Commenting on the importance of the development of national indicators for the assessment of quality, one of the respondents stated that, “All ranking indicators are important, but the problem is we need to weight them according to our needs."

The next sub-theme was the environment. Participants interpreted the environment as the country's contextual conditions affecting the educational sector and the performance of the universities.

“The factors like alumni placement, integration of the industry and universities are important in the context of a developing country like Pakistan."

One participant added to this.

“One of the most important criteria that need to be focused on is the emphasis on the career trajectories and job outcomes after quality education and faculty."

#### 3.1.3 Indicators

Participants discussed indicators of GRS, and those were recategorized according to the Pakistani higher education sector context. Indicators and criteria included and rearranged were taken from the global/national ranking system and participants' input. Following sub-themes emergent in discussion with the participants, where dimensions were treated as “criteria.” and items as “indicators."

##### 3.1.3.1 Quality of teaching/education

Quality of education is considered effective in improving the university's overall performance and provides a healthy learning environment for students. Several indicators were considered adequate and were recommended by the participants for measuring teaching quality; however, other dimensions like “Alumni of institutions winning Nobel Prizes and field medals” were suggested to be discarded. One of the respondents stated that.

“The percentage of Pakistani institutes nominated for global awards, contributions in research and subject development is quite less compared to the other countries, so including this particular factor for evaluating university performance or an indicator for performance isn't adequate to the Pakistani context. We should focus only on related and relevant factors, not those still needing development".

Participants agreed that quality of education must include per capita performance, employers' reputation survey, faculty quality, university facilities, and academic peer reputation:

“The faculty-to-student ratio is important; we have to see how many students there are as per faculty because if students are more, the quality of teaching can be compromised."

One more participant explained:

“If you talk about the quality of education, then the most important element is a teacher. His learning, training and development, research, knowledge on his subject, understanding of his way of teaching, and the quality of the sources being provided to him by his institute that will help him …."

##### 3.1.3.2 Research

The participants selected various research-related factors that needed to be included, like international faculty ratio, student and faculty citations, etc. However, there were some factors that, through mutual consensus of the respondents, were dropped from addiction. Dimensions like research income, volume, income, and reputation were let go because while faculty and student body of Pakistani institutes are gearing towards research, they aren't specifically concerned about the remuneration associated with research as it is an irrelevant factor until now.

Participants agree that it is clear, like a crystal, that HEIs in today's world can only progress and perform well if they keep themselves updated through research.

“Research by students/faculty shows the institution's credibility, including national or international conference paper, HEC or ISI index journal, impact factor journal, and citations per faculty, etc."

Participants reflected on and agreed that many industries fund research projects if they get a solution to their problem, which will be mutually beneficial.

“Funding agencies can also provide you with funds like NRPU (National Research Program for Universities), HEC, etc. But in the industrial sector, the industry has funds, and you have the skill-set, the industry will approach you and ask to give a solution to their problem."

###### 3.1.3.3 Social integration

Participants reflected that social integration had become an essential part of the universities. They suggested various means of communication with stakeholders besides their social media platforms and suggested that indicators like international student and faculty ratio should be included. However, participants suggested that features like international collaboration and international outlook from “THE” shouldn't be included as the factors don't entirely reflect the international integration environment.

“We have to say yes to social integration in this era. Because it showcases the performance of our university

###### 3.1.3.4 Alumni satisfaction

Alumni satisfaction is a significant factor, and participants acknowledged that alumni satisfaction plays a vital role in depicting university performance, provided they get good job opportunities after getting a degree from a particular university:

“We have no issues with our alumni from their employers here formally. I don't think that we've ever received a suggestion that your students are weak."

###### 3.1.3.5 Student satisfaction

Participants believed student satisfaction is an important indicator, but teachers' quality and teaching methods impact the overall performance of the students. They agreed that features like opportunities for career development, grading policies, and research grants were some of the essential features that needed to be incorporated as they would reflect the students' opinions relating to the faculties and universities.

“Student satisfaction is essential. Good teaching is the cause of it. Unfortunately, some students only get satisfied with their grades. But satisfaction, in the long run, is related to the teacher who taught you well, though gave you lesser marks but provided you with knowledge."

Another participant added:

“When you produce good graduates, their subject knowledge is good, and they are performing well in institutes, then they will be selected. It depends on the quality of teaching."

###### 3.1.3.6 Digital presence

Participants agreed that digital presence has become vital for educational institutions in the recent era due to the pandemic. Higher education has mostly became online these past few years, and universities, teachers, and students have struggled immensely to learn digital skills to ensure educational continuity:

The only source today is the internet, i.e., websites. Social media pages are also an option, but no one usually opts. When you want to explore a university, the first source is to check their website. It can include faculty profiles, number of programs, co-curricular activities, etc."

####### 3.1.3.7 Governance

Governance controls the various operations of the HEI, ensuring effective outcomes. “Good Governance University” is considered necessary for balancing “the autonomy granted to institutions as well as accountability.” Participants agreed:

“HEIs are only focusing on the self-assessment report. Governance should be given more weight. So institutional quality can be improved."

Participants suggested proper management, as well as direction, is needed to improve the performance of the universities, which will ultimately promote transparency among various stakeholders.

## 4 Discussion

This study provided valuable insights through the selection of performance indicators for HEIs. The focus group dynamics and participant interaction facilitated a deeper exploration and elucidation of participants' perspectives about ranking systems and the criteria they employ to evaluate national universities. Additionally, participants put forth various indications that might boost institutions' enhancement and long-term viability. The significance of this work lies in its unique exploration of ranking indicators within the specific context of Pakistan, employing a methodology that has not been utilized before to investigate these questions. This study aimed to identify fundamental components necessary for establishing a ranking framework, which was successfully achieved via the focus groups as explained in the previous section.

The importance of these factors has been highlighted by previous research, Çakır et al. [34] suggest that Pakistan and China focus on size-independent indicators for ranking, similar to global ranking systems such as THE and QS. However, the study suggests that only one global ranking system, URAP, includes the ranking of very few Pakistani institutes. The present study elaborates that the Pakistani ranking system assigns high weightage to implementing quality assurance criteria, teaching quality, and research. Qazi et al. [35] suggested that Pakistani institutions focus on research articles, citations, and total documents (related to scientific productivity) for the university's performance regarding research. The current study also supports the opinion that indicators related to research and quality of education should be given more weightage or implemented to increase the productivity of students and faculty so they can match international standards.

Shehatta et al. [36] contends that the Pakistani ranking system is like URAP and Webometrics ranking system, encompassing research factors like times cited, citation impact, and impact relative to the world. The study finds that the ranking system is reliable despite previous criticisms and can measure performance. As per the present study's findings, Pakistan needs improvement to indicate the performance of universities correctly. Quality of education is considered effective in improving a university's overall performance; it ensures a healthy learning environment for the students and helps them gain higher education, effectively getting better employment [37,38]. While discussing the impact of the quality of education on university performance, the participants agreed that per capita performance, employers' reputation survey, faculty quality, university facilities (Research tools, Libraries, Labs, etc.), and academic peer reputation survey are critical indicators to evaluate the quality of education. Participants agreed to research another criterion to rank HEIs [39]. Henry et al. [40] and Nafukho et al. [40,41] suggested for a productive and innovative learning environment, the universities should give them essential research tools, other grants related to research, and travel. Participants agreed to travel grants, Ph.D./total students, H-Index, Number of Citations per paper, Research Productivity, and Industry linkages as indicators to measure research.

Khalid et al. [3] studied the quality of higher education and found that it can be improved by creating internationalized curricula, hiring international students and faculty, and forming international research collaboration projects. Higher education institutions in Pakistan must rethink and transform internationalization strategies per global standards, which would benefit the seamless transition to an international knowledge social structure. The current study furthers the previous research and increases knowledge by highlighting these factors in social integration.

Social integration has helped many universities attract international students and faculty [42,43]. Outreach programs, collaboration/exchange, foreign faculty ratio to all faculty, and international students' ratio to all students are the agreed parameters to check social integration. Furthermore, alumni satisfaction needs to be aimed by the universities to improve the overall image of the institute [44,45]. Indicators of alumni satisfaction include satisfaction with Career Trajectory, employment opportunities, and commitment to the institute. Participants agreed that student satisfaction, which can be measured through career development opportunities, satisfaction with the research budget, satisfaction with grading, and plagiarism policies, is another university ranking criterion.

Higher education has mostly become online these past few years, and universities, teachers, and students have struggled immensely to learn digital skills to ensure educational continuity [46,47]. Visibility, transparency, and excellence of the university website, number of backlinks, and number of referring domains are considered necessary while evaluating digital presence are recommended by literature.

Finally, governance is supported by the literature Kurniawati et al. [48] and Sulila [49] highlighted that governance is essential in ensuring university quality. It controls the various operations of the university, ensuring effective outcomes. Management and direction, accountability, transparency, and autonomy are governance indicators. These findings are aline with Aziz et al. [50], who indicated that the government has failed to implement an effective system for the development and performance of Pakistani education. The focus on infrastructure should increase, including factors like better governance, financing, human resources, and curriculum, to expect better performance of HEIs. Thirty indicators divided into seven criteria’ are mentioned in (Table 4).

**Table 4:** Final set of criteria and indicators.

Criteria	Indicators
Quality of Education	Per Capita Performance
	Employers' reputation survey
	Faculty Quality
	University Facilities
	Academic peer reputation survey
Research	Travel Grants
	Ph.D./total students
	H Index
	Number of Citations per paper
	Research Productivity
	Research funding by the industrial sector
Social Interaction	Outreach programs
	Collaboration/Exchange
	Foreign Faculty Ratio to all Faculty
	International Students' ratio to all Students
Alumni Satisfaction	Satisfaction with Career Trajectory
	Satisfaction with Employment Opportunities
	Satisfaction with Commitment
Student Satisfaction	Career Development Opportunities
	Satisfaction with Research Budget
	Satisfaction with Grading and Plagiarism Policies
Governance	Management and Direction
	Accountability
	Transparency
	Autonomy
Digital presence	Visibility
	Transparency
	Excellence
	Number of backlinks
	Number of referring domains

## 5 Implications

### 5.1 Theoretical implications

The study contributes to the overall theoretical development and understanding of the role played by ranking systems and their effect on university performance. The study also contributes valuably to the literature focusing on regional-specific indicator governance, which will create transparency in university administration.

### 5.2 Managerial implications

The accurate interpretation and use of these criteria are essential for university administration to help them improve performance. Regulators can allocate resources to national institutions based on the performance of HEIs, particularly in the research and development areas, leading to more effective utilization of funds and alignment with the national educational outlook. Furthermore, students could discern universities based on their respective academic achievements.

## 6 Limitations

This study's limitations include using purposive sampling, which is prone to the selection bias. The frequency of the focus groups, mode of conduction, and sample size were small, and all the participants were from Pakistan; findings may not be generalized in another contextual setting. Another possible limitation of the present study was that the data was collected only from HEC/QEC staff, faculty members (university employees), and doctoral scholars. The study is also limited from the perspective of the method used for investigating the research problem, which can be diversified in the future.

## 7 Conclusions and recommendations

This study aimed to examine a selection of indicators most appropriate for assessing the institutional performance of Pakistani higher education institutions (HEIs). Focus groups were employed in the study to gather diverse perspectives, facilitate in-depth discussions, and enrich the qualitative data, thereby enhancing the comprehensiveness and depth of the research findings. The focus groups analyzed global and national ranking systems to identify indicators and criteria that enhance university performance. The seven selected criteria include the quality of education, research, social interaction, alumni satisfaction, student satisfaction, governance, and digital presence. None of the global ranking systems contained governance, and participants emphasized the necessity of its inclusion to address local requirements of transparency, accountability, and better decision-making while assessing risk and promoting long-term sustainability. Findings propose the potential development of an empirically tested ranking system that might address the challenge of evaluating the performance and precise goal-setting of HEIs. Future research might use different sampling procedures for a more thorough and inclusive analysis, including international participation and perspective. Combining quantitative methods with focus groups in future studies will ensure a more comprehensive understanding by quantifying data and gaining qualitative insights from participants. Future research should also investigate the correlation between improvement in rankings and real enhancements of a university's performance.

## Ethics statement

This research did not involve any experiment that might include human or animals. Therefore, this research did not harm any human or animal in any experimentation. All the ethical standards of American Psychological Association (APA) were followed by the study which were applicable to the qualitative research. The data was taken from the focus group whose informed consent was taken on written consent form duly signed by them. There was no need to get permission of institutional review board (IRB) because the members of focus group provided their free consent and acted in their personal capacities. Moreover, the confidentiality of the data was made sure. There was no coercion, no injustice, nor anything else was involved that might harm the focus group mentally, physically, or psychologically.

## Funding statement

This research received no financial grant from any funding agencies in the public, commercial, or not-for-profit sectors.

## Additional information

Any additional information required by the journal would be provided.

## Data availability statement

Data included in article/supp. Material/referenced in article.

## CRediT authorship contribution statement

**Tayyaba Rafique:** Writing – review & editing, Writing – original draft, Methodology, Formal analysis, Conceptualization. **Muhammad Usman Awan:** Validation, Supervision, Conceptualization. **Muhammad Shafiq:** Validation, Supervision, Data curation. **Khalid Mahmood:** Conceptualization.

## Declaration of competing interest

The authors declare that they have no known competing financial interests or personal relationships that could have appeared to influence the work reported in this paper.
